# Deoxyribose Nucleic Acid Damage and Its Association With Plasma Malondialdehyde Levels Among Patients With Cervical Cancer: A Case-Control Study

**DOI:** 10.7759/cureus.52600

**Published:** 2024-01-19

**Authors:** Sankara Narayanan G, Rajasekhar SSSN, Latha Chaturvedula, Prashant Adole

**Affiliations:** 1 Anatomy, Saveetha Medical College and Hospital, Chennai, IND; 2 Anatomy, Jawaharlal Institute of Postgraduate Medical Education and Research, Puducherry, IND; 3 Obstetrics and Gynaecology, Jawaharlal Institute of Postgraduate Medical Education and Research, Puducherry, IND; 4 Biochemistry, Jawaharlal Institute of Postgraduate Medical Education and Research, Puducherry, IND

**Keywords:** electrophoresis, plasma malondialdehyde, dna damage, comet assay, cervical cancer

## Abstract

Purpose

The objective of this research project was to estimate DNA damage in patients diagnosed with cervical cancer using the comet assay, establish a correlation between this quantification and the oxidative stress marker malondialdehyde (MDA; plasma MDA), and compare the resulting parameters between the cases and age-matched controls.

Materials and methods

This study included 49 cervical cancer cases and 49 age-matched controls to measure DNA damage parameters such as comet length, head diameter, percentage of DNA in the comet head, tail length, percentage of DNA in the comet tail, and oxidative stress marker (plasma MDA) using the thiobarbituric acid reactive substance (TBARS) enzyme-linked immunosorbent assay (ELISA) method.

Results

Comet metrics suggesting DNA damage, such as comet length, tail length, and percentage of DNA in the comet tail, were considerably higher in cervical cancer cases than in age-matched controls. The proportion of DNA in the comet head, representing undamaged/mild DNA damage, was significantly higher in age-matched controls than in cervical cancer patients. Plasma MDA and comet tail length were shown to have a positive correlation. Compared to the age-matched controls, those between the ages of 30 and 39, with a parity of two to four, who had a history of early age at first pregnancy and a positive family history of cervical cancer, had the highest level of DNA damage.

Conclusion

The elevated levels of comet parameters and their positive correlation with plasma MDA suggest that individuals diagnosed with cervical cancer have a higher degree of DNA damage compared to the control group. In conjunction with established methods like the PAP smear, this predictive test comprising comet assay and estimation of plasma MDA may be utilized to identify and assess the risk of cervical cancer in individuals aged 30-39 years, with a parity between two and four pregnancies and a prior history of early age at first pregnancy, accompanied by a positive family history of the disease.

## Introduction

Burden

**Figure 1:**
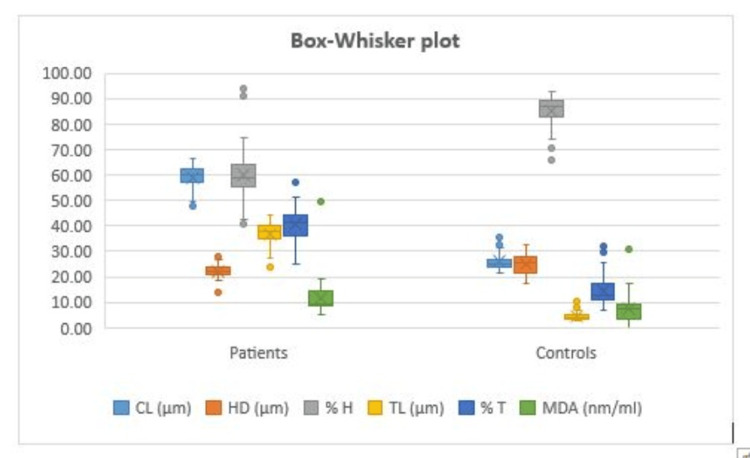
Box and Whisker plot showing the comparison of mean comet parameters and plasma malondialdehyde (MDA) in cervical cancer cases and control. p-value <0.05 was considered as statistically significant. CL: comet length, HD: comet head diameter, %H: percentage of DNA in the comet head, TL: comet tail length, %T: percentage of DNA in the comet tail, MDA: plasma malondialdehyde.

Based on 2018 WHO figures [1], cervical cancer ranks as the fourth most prevalent cancer globally. Additionally, global cancer statistics [2,3] place it as the fourth leading cause of cancer-related fatalities worldwide, accounting for 7.5% of cases. In India in 2018, cervical cancer ranks as the third most prevalent malignancy and the second leading cause of death among cancer-related diseases. In Tamil Nadu, it ranks second in terms of cancer-related fatalities, accounting for 22.5% [2,4]. A 20% increase in the incidence of cervical cancer in India compared to the incidence documented in 2010 is anticipated by the year 2020 [5]. Cervical cancer, unlike other malignancies, typically manifests in women during their early years, namely within the age range of 21-67 years [6].

Risk factors

Multiple sexual partners, poor genital hygiene, HIV, human papillomavirus (HPV), chlamydia, the use of oral contraceptives, intrauterine devices, diethylstilboestrol, oral contraceptives, and smoking, premature or late pregnancies, and multiple full-term pregnancies are all risk factors for cervical cancer [7-11].

HPV infection and DNA damage

Among the several risk factors, human papillomavirus (HPV) infection is regarded as the most significant, accounting for 99% of cervical cancer cases globally [12]. Cervical cancer has been linked to carcinogenic strains, including HPV 16, 18, 31, and 33 [13]. In HPV infections, the viral integrated host genome secretes oncoproteins, including E6 and E7, disrupting the regular cell cycle. This disruption leads to the production of damaged or mutant deoxyribose nucleic acid (DNA) in greater quantities [14-18], resulting in genomic instability. Cervical cancer arises from the monoclonal proliferation of cells in the epithelium, induced by an elevated degree of genetic instability [14-18]. It is possible to quantify the extent of DNA damage from the nucleus of circulating lymphocytes [19]. Determining the extent of DNA damage at the level of a single cell is thus crucial. Comet assays or single-cell gel electrophoresis (SCGE) are used to identify alkali-labile sites and single-stranded breaks. This method is simple, inexpensive, rapid, and requires less than one hundred cells, making it an excellent genetic screening test.

Lipid peroxidation

Lipid peroxidation during HPV infections generates reactive oxygen species or free radicals in excess, both byproducts of cellular metabolism. The equilibrium between reactive oxygen species and antioxidant levels within the cell is critical for cellular processes such as DNA replication, protein synthesis, and division [20-22]. An increase in free radical production causes an imbalance between free radicals and antioxidants, leading to an oxidative stress response, which damages the deoxyribose and nucleotide bases. The production of the E6 oncoprotein is associated with an elevation in oxidative stress during HPV infections, leading to lipid peroxidation [23]. HPV infection significantly influences the DNA damage response (DDR), a critical factor in the advancement of cervical cancer [24]. Abnormal cell replication and defective DNA repair result in cellular demise or malignancy [25,26].

Malondialdehyde (MDA)

Primarily, lipid peroxidation generates malondialdehyde (MDA), considered the international standard biomarker for lipid peroxidation in plasma. Hydroperoxide is generated when phospholipids are subjected to stress; subsequently, hydroperoxy-aldehyde is produced by β-cleavage of fatty acid chains. The released MDA contributes to the progression of the illness by causing harm to cellular organelles and the nuclear membrane [27-30]. Assessing oxidative stress and lipid peroxidation using MDA estimates is straightforward, practical, economical, and user-friendly [23,31,32]. Assigning the degree of oxidative stress to plasma MDA to detect cervical cancer cases was proposed as a potential screening method [33]. Alongside existing methods including visual inspection by acetic acid (VIAA), visual inspection by Lugol's iodine (VILI), and Papanicolaou's smear (PAP), comet assay has the potential to quantify the extent of DNA damage. This would enable these methods to be utilized as predictive tests for early detection and risk assessment of cervical cancer in the most susceptible demographics [34].

When it comes to a disease that is as prevalent as cervical cancer, there is a shortage of published literature that discusses the role that genetics plays. In instances of cervical cancer, the purpose of the current investigation was to determine if DNA damage detected at the first diagnosis may serve as a screening or prognostic test. As the cancer advances, the objective of the current investigation was not to quantify the extent of DNA damage at different stages. Plasma MDA and comet assay measurements, respectively, were used in the current investigation to determine the amount of oxidative stress and DNA damage in newly diagnosed cases of cervical cancer before treatment began. In addition, the correlation between plasma MDA concentrations and DNA damage in instances of cervical cancer is assessed.

This article was previously posted to the Research Square preprint server on April 26, 2022, with DOI: https://doi.org/10.21203/rs.3.rs-269996/v1.

## Materials and methods

The current investigation was conducted at the cytogenetics laboratory of the Department of Anatomy at JIPMER, Puducherry, India, in conjunction with the Department of Obstetrics & Gynecology and the Department of Biochemistry. The research received approval from both the Departmental Postgraduate Research Monitoring Committee (PGRMC) and the Institute Human Ethics Committee (Ref No: JIP/IEC/2017/0370, Dated 05.04.2017).

Study population

Study Group One: 49 Cases of Cervical Cancer

Inclusion criteria included female patients willing to participate in the study, newly diagnosed cervical cancer patients before starting treatment, and clinically and histopathologically (biopsy) proven cancer patients before starting treatment. The age range of patients in study group one was 36-82 years, and the parity range was 0-6. Exclusion criteria included cases of cervical cancer already undergoing treatment, cases of cervical cancer accompanied by concomitant problems like hypertension, diabetes mellitus, renal, hepatic, thyroid, or lung diseases, other conditions such as autoimmune illnesses and known genetic disorders; pregnant and lactating women; and those who were not willing to participate in the study.

Study Group Two: 49 Age-Matched Controls

Inclusion criteria included female patients visiting the Gynaecology OPD for minor illnesses willing to participate in the study, and those with a negative Pap smear for intraepithelial cancers. The age range of patients in study group two was 30-80 years, and the parity range was 0-6. Exclusion criteria included any other kind of cancer, hypothyroidism, diabetes mellitus, and other conditions such as autoimmune illnesses and known genetic disorders; pregnant and lactating women; and those who were not willing to participate in the study.

Sample size calculation

The sample size of 49 newly diagnosed cervical cancer cases and 49 age-matched controls was determined using PASS software, version 3.1.2, to compare the two means. With a standard deviation of 3.5, the least predicted difference in tail length between groups was two. The sample size was calculated using a 5% threshold of significance and an 80% power level. The sample size was estimated using prior research estimations of all other study parameters. A larger sample size was suggested as the difference in tail length results in a larger sample size [34].

List of variables observed in the study

Independent factors noted were family history, number of pregnancies, age at first pregnancy, smoking history, alcohol consumption history, and sexual history. The outcome variables assessed were plasma MDA and comet characteristics such as comet length, head diameter, percentage of DNA in the head, tail length, and percentage of DNA in the tail. There were no interfering or confounding factors.

Methodology

The current study is divided into two procedures: (a) DNA damage analysis was carried out using the comet assay method [35]. (b) MDA levels in plasma were determined using TBARS (ELISA) techniques.

Estimation of plasma MDA using the TBARS (ELISA) method

After purchasing the TBARS test kit, it was stored in the freezer at 2-8°C. Following blood collection from each patient, the blood was centrifuged to separate cells from plasma. The cells were used in the comet assay process, and the plasma was stored in the freezer at -80°C.

To analyze plasma MDA, the plasma was removed from the freezer six hours before the MDA-TBARS test process to allow thawing. Similarly, the TBARS test kit was taken out of the freezer and brought to room temperature. All of the standard solutions and working reagents were prepared from the kit according to the procedure included in the kit. The number of strips needed for the test was determined and placed in the frames for use. The unused strips were stored in the freezer at 2-8°C for future use. A standard well received 50 µL of the standard solution, whereas sample wells received 40 µL of samples and 10µl of anti-MDA antibody. Then, 50 µL of streptavidin-HRP was added to each sample and control well (not blank control well). The mixture was well mixed, and the plate was sealed and incubated at 37°C for 60 minutes. After removing the seal, the plate was washed five times with 0.35 mL of wash buffer, with each wash lasting 30 seconds to 1 minute. Paper towels were used to blot the plate. Fifty microliters of substrate solutions A and B were added to each well. The plate was then incubated for 10 minutes in the dark at 37°C with a fresh sealer. Following the incubation period, 50 µL of stopping solution was added. The color shift from blue to yellow was quickly noticed. The microplate reader was adjusted to 450 nm to analyze the optical density value from each well. The plate was transferred to the microplate reader for optical density measurement within 10 minutes after the color change. The OD value released by each well represents the amount of MDA in patients' and controls' plasma.

Statistical analysis

The frequency and percentage distributions of categorical factors such as family history, smoking history, alcohol consumption history, and DNA damage were calculated. Age, number of pregnancies, age at first pregnancy, amount of DNA damage, and plasma MDA level were reported as means with standard deviations for both discrete and continuous variables. An independent Student's t-test was used to compare the levels of DNA damage and plasma MDA across groups. Correlation analysis was used to determine the connection between DNA damage and plasma MDA levels. All analyses were performed using a 5% significance threshold; p < 0.05 was deemed significant. The data obtained from the study were entered into a Microsoft Excel spreadsheet (Microsoft Corporation, Redmond, Washington) for descriptive analysis.

## Results

A total of 49 new cervical cancer patients and 49 age-matched controls participated in the study and were tested for DNA damage and plasma MDA levels.

Comparison of comet parameters and plasma MDA in cervical cancer cases and controls

Comet metrics such as comet length, comet tail length, percentage of DNA in the comet tail, and plasma MDA levels were considerably higher in cervical cancer patients compared to controls. When compared to cervical cancer cases, comet metrics such as head diameter and percentage of DNA in the head were considerably greater in controls (Table 1).

**Table 1:** Comparison of mean comet parameters and plasma malondialdehyde(MDA) in cervical cancer cases and control. * p-value <0.05 was considered as statistically significant.

S. No.	Parameters	Patients	Controls	p-value
		Mean ± SD	Mean ± SD	
1	Comet length (µm)	58.96 ± 4.88	26.22 ± 3.34	0.001^*^
2	Comet head diameter (µm)	22.33 ± 2.49	24.75 ±3.22	0.001^*^
3	Percentage of DNA in the comet head (%)	60.30 ± 9.76	85.37 ± 6.06	0.001^*^
4	Comet tail length (µm)	36.76 ± 5.16	4.65 ± 1.93	0.001^*^
5	Percentage of DNA in the comet tail (%)	40.81 ± 7.25	14.63 ± 6.06	0.001^*^
6	Plasma malondialdehyde level (nmol/L)	11.62 ± 6.53	7.75 ± 5.60	0.001^*^

We analyzed these comet characteristics and plasma MDA by categorizing research participants based on age, parity, number of pregnancies, risk factors, and cancer family history. Box-Whisker plot has been shown for statistical analysis in Figure 1.

Age group-wise comparison of comet parameters and plasma MDA levels

Across all age categories, we found that comet length was longer in cervical cancer cases than in controls. Comet duration in cervical cancer cases was greatest in the 60-year age group, followed by 30-39 years, 40-49 years, and 50-59 years (Table 2).

**Table 2:** Age group-wise comparison of the comet parameters and plasma malondialdehyde (MDA) in cervical cancer cases and controls. All the parameters are expressed in mean ± SD.

Groups (based on the age of the participants)		30-39 years	40-49 years	50-59 years	≥60 years
Comet length (µm)	Cervical cancer cases (n=49)	59.15 ± 4.39 (n=3)	58.52 ± 5.99 (n=9)	56.08 ± 10.90 (n=19)	59.89 ± 4.81 (n=18)
	Controls (n=49)	26.11 ± 2.69 (n=27)	27.54 ± 5.12 (n=12)	24.87 ± 1.59 (n=9)	25.37 ± 0 (n=1)
Head diameter (µm)	Cervical cancer cases (n=49)	19.43 ± 4.62 (n=3)	23.4 ± 2.37 (n=9)	22.18 ± 2.51 (n=19)	22.44 ± 1.88 (n=18)
	Controls (n=49)	21.38 ± 2.11 (n=27)	23.45 ± 5.33 (n=12)	22.81 ± 1.80 (n=9)	22.68 (n=1)
Percentage of DNA in the comet head (%)	Cervical cancer cases (n=49)	59.64 ± 3.41 (n=3)	64.08 ± 13.02 (n=9)	66.17 ± 10.55 (n=19)	60.58 ± 7.30 (n=18)
	Controls (n=49)	84.37 ± 6.70 (n=27)	85.78 ± 5.71 (n=12)	87.60 ± 4.45 (n=9)	87.19 (n=1)
Tail length (µm)	Cervical cancer cases (n=49)	39.72 ± 1.44 (n=3)	35.85 ± 6.30 (n=9)	36.05 ± 5.14 (n=19)	37.45 ± 5.00 (n=18)
	Controls (n=49)	4.78 ± 2.12 (n=27)	4.58 ± 1.98 (n=12)	4.29 ± 1.44 (n=9)	4.86 ± 0 (n=1)
Percentage of DNA in the comet tail (%)	Cervical cancer cases (n=49)	40.35 ± 3.41 (n=3)	40.24 ± 8.31 (n=9)	40.11 ± 7.30 (n=19)	42.41 ± 7.30 (n=18)
	Controls (n=49)	15.62 ± 6.0 (n=27)	14.21 ± 5.71 (n=12)	12.39 ± 4.45 (n=9)	12.80 ± 0 (n=1)
Plasma malondialdehyde level (nmol/L)	Cervical cancer cases (n=49)	24.22 ± 22.00 (n=3)	11.18 ± 4.17 (n=9)	11.71 ± 3.73 (n=19)	9.64 ± 2.86 (n=18)
	Controls (n=49)	7.94 ± 4.26 (n=27)	8.57 ± 5.27 (n=12)	6.68 ± 9.20 (n=9)	2.35 ± 0 (n=1)

Across all age groups, controls had larger heads than cervical cancer patients. The age group of 40-49 years had the largest head diameter in cervical cancer instances, followed by 60 years, 50-59 years, and 30-39 years. Across all age categories, the proportion of DNA in comet heads was greater in controls than in cervical cancer cases. The proportion of DNA in the comet head in cervical cancer cases was greater in the age range of 50-59 years, followed by 40-49 years, 60 years, and 30-39 years. In all age groups, cervical cancer patients had longer tails than controls. The age group of 30-39 years had the longest tail length in cervical cancer cases, followed by 60 years, 50-59 years, and 40-49 years. Across all age categories, the proportion of DNA in the comet tail was greater in cervical cancer cases than in controls. In cervical cancer cases, the proportion of DNA in the comet tail was highest in the 60-year age group, followed by the 30-39-year, 40-49-year, and 50-59-year age groups. The plasma MDA level in cervical cancer patients was greater than in controls across all age groups. In cervical cancer patients, plasma MDA levels were highest in those aged 30-39 years, followed by those aged 50-59 years, 40-49 years, and 60 years (Table 2).

Effect of parity

Comet length was higher in different parity groups of cervical cancer cases compared to controls. In cervical cancer cases, the comet length was higher in the parity 2-4 followed by the parity ≥5 and parity 0-1. However, no samples were available for the study in the parity group ≥5, in controls (Table 3).

**Table 3:** Parity-wise comparison of the comet parameters and plasma malondialdehyde in cervical cancer cases and controls. All the parameters were expressed in mean ± SD.

Parity groups (based on the number of pregnancies)		0-1	2-4	≥5
Comet length (µm)	Cervical cancer cases (n=49)	57.78 ± 0 (n=1)	58.15 ± 8.22 (n = 42)	57.94 ± 6.71 (n = 6)
	Controls (n=49)	27.49 ± 5.61 (n = 4)	26.10 ± 3.14 (n = 45)	0 (n = 0)
Head diameter (µm)	Cervical cancer cases (n=49)	26.52 ± 0 (n=1)	22.26 ± 2.43 (n=42)	22.34 ± 6.71 (n=6)
	Controls (n=49)	24.27 ± 5.53 (n=4)	21.52 ± 2.93 (n=45)	0 (n = 0)
Percentage of DNA in the comet head (%)	Cervical cancer cases (n=49)	60.29 ± 0 (n=1)	60.16 ± 10.47 (n=42)	61.58 ± 3.25 (n=6)
	Controls (n=49)	87.16 ± 1.62 (n=4)	85.21 ± 6.29 (n=45)	0 (n = 0)
Tail length (µm)	Cervical cancer cases (n=49)	31.26 ± 0 (n=1)	37.02 ± 5.22 (n=42)	35.80 ± 4.87 (n=6)
	Controls (n=49)	3.68 ± 1.07 (n=4)	4.73 ± 1.97 (n=45)	0 (n = 0)
Percentage of DNA in the comet tail (%)	Cervical cancer cases (n=49)	41.71 ± 0 (n=1)	41.92 ±7.70 (n=42)	38.42 ± 3.26 (n=6)
	Controls (n=49)	12.83 ± 1.67 (n=4)	14.78 ± 6.29 (n=45)	0 (n = 0)
Plasma malondialdehyde level (nmol/L)	Cervical cancer cases (n=49)	17.84 ± 0 (n=1)	21.90 ± 6.88 (n=42)	18.62 ± 1.42 (n=6)
	Controls (n=49)	12.77 ± 4.22 (n=4)	7.30 ± 5.52 (n=45)	0 (n = 0)

When comparing cervical cancer cases to controls, the head width was larger in various parity groups. The head diameter in cervical cancer patients was greater in parity 0-1, followed by parity 5, and parity 2-4. When compared to cervical cancer cases, the proportion of DNA in comet heads was greater in various parity groups of controls. The proportion of DNA in the comet head in cervical cancer patients was greater in the parity group 5, followed by the parity groups 0-1 and 2-4. However, no samples were obtained for the investigation in the parity group 5 among controls. The length of the comet tail was longer in various parity groups of cervical cancer patients than in controls. The comet tail length in cervical cancer cases was greater in parity groups 2-4, followed by parity groups 5 and 0-1. However, no samples were obtained for the investigation in the parity group 5 among controls. The DNA percentage in the comet tail of cervical cancer cases was greater in various parity groups than in controls. The proportion of DNA in the comet tail in cervical cancer patients was highest in the parity groups 2-4, followed by the parity groups 0-1 and 5. However, no samples were obtained for the investigation in the parity group 5 among controls. When comparing cervical cancer patients to controls, plasma MDA levels were higher in various parity groups. Plasma MDA levels in cervical cancer patients were greater in parity groups 2-4, followed by parity groups 5 and 0-1. However, no samples were obtained for the investigation in the parity group 5 among controls (Table 3).

Effects of pregnancy at an early age

Comet length was longer in cervical cancer patients (age at first pregnancy 19 years and older) than in controls. The comet length was greater in cervical cancer patients with a first pregnancy age of 19 years compared to >19 years (Table 4).

**Table 4:** Comparison of comet parameters in cervical cancer cases and controls according to the age at first pregnancy. All the parameters were expressed in mean ± SD.

Groups (based on the age at first pregnancy)		Age at first pregnancy ≤19 years	Age at first pregnancy >19 years
Comet length (µm)	Cervical cancer cases (n=49) (Nil = 1)	59.94 ± 9.74 (n=28)	59.49 ± 4.40 (n=20)
	Controls (n=49) (Nil = 2)	25.46 ± 2.96 (n=15)	26.71 ± 3.52 (n=32)
Head diameter (µm)	Cervical cancer cases (n=49) (Nil = 1)	22.72 ± 1.77 (n=28)	22.55 ± 3.18 (n=20)
	Controls (n=49) (Nil = 2)	21.44 ± 3.01 (n=15)	21.90 ± 3.44 (n=32)
Percentage of DNA in the comet head (%)	Cervical cancer cases (n=49) (Nil = 1)	60.38 ± 11.88 (n=28)	60.78 ± 6.29 (n=20)
	Controls (n=49) (Nil = 2)	87.53 ± 3.89 (n=15)	84.19 ± 6.76 (n=32)
Tail length (µm)	Cervical cancer cases (n=49) (Nil = 1)	37.58 ± 5.73 (n=28)	37.26 ± 4.31 (n=20)
	Controls (n=49) (Nil = 2)	4.27 ± 1.16 (n=15)	4.93 ± 2.19 (n=32)
Percentage of DNA in the comet tail (%)	Cervical cancer cases (n=49) (Nil = 1)	41.55 ± 8.00 (n=28)	39.71 ± 6.29 (n=20)
	Controls (n=49) (Nil = 2)	12.46 ± 3.89 (n=15)	15.80 ± 6.76 (n=32)
Plasma malondialdehyde level (nmol/L)	Cervical cancer cases (n=49) (Nil = 1)	10.99 ± 3.51 (n=28)	12.19 ± 9.34 (n=20)
	Controls (n=49) (Nil = 2)	6.11 ± 7.81 (n=15)	8.35 ± 4.27 (n=32)

Both types of cervical cancer patients (age at first pregnancy 19 years and >19 years) had larger head diameters than controls. The head diameter was larger in cervical cancer patients with a first pregnancy age of 19 years compared to >19 years. When compared to cervical cancer cases, the proportion of DNA in the comet head was greater for control groups (age at first pregnancy 19 years and >19 years). The proportion of DNA in the comet head was greater in cervical cancer patients with a first pregnancy age of more than 19 years compared to 19 years. The length of the comet tail was longer in cervical cancer patients (age at first pregnancy 19 years and >19 years) than in controls. The comet tail length was longer in cervical cancer patients with a first pregnancy age of 19 years compared to >19 years. The proportion of DNA in the comet tail was greater in cervical cancer cases than in controls (age at first pregnancy 19 years and >19 years). The proportion of DNA in the comet tail was greater in cervical cancer patients with a first pregnancy age of 19 years compared to >19 years. Plasma MDA was greater in cervical cancer patients (age at first pregnancy 19 years and >19 years) than in controls. Plasma MDA was greater in cervical cancer patients with an age at first pregnancy of >19 years compared to 19 years (Table 4).

Effects of risk factors

The comet length was longer in cervical cancer cases (with 1 or more risk factors) than in controls. The comet length was greater in cervical cancer patients with more than one risk factor (Table 5).

**Table 5:** Comparison of comet parameters in cervical cancer cases and controls according to risk factors. Risk factor 1 = early pregnancy, Risk factor 2 = dysmenorrhea, Risk factor 3 = family history of any cancer, Risk factor 4 = alcohol intake, Risk factor 5 = cigarette smoking. All the parameters were expressed in mean ± SD.

Groups (based on the number of risk factors in the pregnant mother)		≤1 Risk factor	> 1 Risk factor
Comet length (µm)	Cervical cancer cases (n=49)	57.69 ± 5.55 (n=14)	58.28 ± 8.73 (n= 35)
	Controls (n=49)	26.37 ± 3.49 (n=43)	25.07 ± 1.73 (n=6)
Head diameter (µm)	Cervical cancer cases (n=49)	24.15 ± 2.85 (n=14)	25.60 ± 1.93 (n=35)
	Controls (n=49)	21.78 ± 3.41 (n=43)	21.47 ± 1.23 (n=6)
Percentage of DNA in the comet head (%)	Cervical cancer cases (n=49)	61.96 ± 6.14 (n=14)	59.63 ± 10.88 (n=35)
	(n=49)	85.06 ± 6.23 (n=43)	87.60 ± 4.37 (n=6)
Tail length (µm)	Cervical cancer cases (n=49)	34.01 ± 5.84 (n=14)	37.85 ± 4.48 (n=35)
	Controls (n=49)	4.75 ± 1.99 (n=43)	3.89 ± 1.27 (n=6)
Percentage of DNA in the comet tail (%)	Cervical cancer cases (n=49)	38.04 ± 6.14 (n=14)	41.91 ± 7.44 (n=35)
	Controls (n=49)	14.93 ± 6.23 (n=43)	12.39 ± 4.37 (n=6)
Plasma malondialdehyde level (nmol/L)	Cervical cancer cases (n=49)	11.92 ± 4.39 (n=14)	11.78 ± 7.26 (n=35)
	Controls (n=49)	7.63 ± 4.61 (n=43)	8.57 ± 11.03 (n=6)

The head diameter was larger in cervical cancer cases (with 1 or more risk factors) than in controls. The head diameter was larger in cervical cancer patients with more than one risk factor. When compared to cervical cancer cases, the proportion of DNA in the comet head was greater in both groups of controls (one or more risk factors). The proportion of DNA in the comet head was greater in cervical cancer patients with just one risk factor. The comet tail length was longer in cervical cancer cases (with 1 or more risk factors) than in controls. The comet tail length was longer in cervical cancer patients with more than one risk factor. The proportion of DNA in the comet tail was greater in cervical cancer cases (with 1 or more risk factors) than in controls. The proportion of DNA in the comet tail was greater in cervical cancer patients with more than one risk factor. Plasma MDA levels were greater in cervical cancer cases (with 1 or more risk factors) than in controls. The plasma MDA in cervical cancer cases was greater in those with one risk factor (Table 5).

Effect of family history of cancer

A positive family history of cervical cancer was found in 43% of cases and 6% of controls. The comet length was longer in cervical cancer cases (both positive and negative family history of cancer) than in controls. The length of the comet was longer in cervical cancer patients with a positive family history of malignancy (Table 6).

**Table 6:** Comparison of comet parameters in cervical cancer cases and controls according to family history of cancer. All the parameters were expressed in mean ± SD.

Groups (based on the presence of family history of cancer)		Positive family history of cancer	Negative family history of cancer
Comet length (µm)	Cervical cancer cases (n=49)	58.48 ± 4.76 (n=21)	57.84 ± 9.70 (n=28)
	Controls (n=49)	24.36 ± 0.53 (n=3)	26.34 ± 3.41 (n=46)
Head diameter (µm)	Cervical cancer cases (n=49)	22.44 ± 2.70 (n=21)	22.40 ± 2.37 (n=28)
	Controls (n=49)	21.81 ± 0.75 (n=3)	21.74 ± 3.32 (n=46)
Percentage of DNA in the comet head (%)	Cervical cancer cases (n=49)	61.94 ± 12.26 (n=21)	59.06 ± 7.36 (n=28)
	Controls (n=49)	86.91 ± 6.38 (n=3)	85.27 ± 6.09 (n=46)
Tail length (µm)	Cervical cancer cases (n=49)	37.24 ± 5.56 (n=21)	37.14 ± 4.89 (n=28)
	Controls (n=49)	2.92 ± 0.35 (n=3)	4.76 ± 1.94 (n=46)
Percentage of DNA in the comet tail (%)	Cervical cancer cases (n=49)	40.64 ± 7.28 (n=21)	40.13 ± 7.36 (n=28)
	Controls (n=49)	13.08 ± 6.38 (n=3)	14.72 ± 6.09 (n=46)
Plasma malondialdehyde level (nmol/L)	Cervical cancer cases (n=49)	12.09 ± 9.20 (n=21)	11.27 ± 3.57 (n=28)
	Controls (n=49)	4.55 ± 2.21 (n=3)	7.96 ± 5.70 (n=46)

The head diameter was larger in cervical cancer cases (both positive and negative family history of cancer) than in controls. The head diameter of cervical cancer patients with a positive family history of cancer was greater. In both groups of controls (positive and negative family history of cancer), the proportion of DNA in the comet head was greater than in cervical cancer cases. The proportion of DNA in the comet head was greater in cervical cancer patients with a positive family history of cancer. The length of the comet tail was longer in cervical cancer cases (both positive and negative family history of cancer) than in controls. The length of the comet tail was longer in cervical cancer patients with a positive family history of cancer. In both groups of cervical cancer patients (positive and negative family history of cancer), the proportion of DNA in the comet tail was greater than in controls. The proportion of DNA in the comet tail was greater in cervical cancer patients who had a positive family history of cancer. Plasma MDA levels were greater in cervical cancer patients (both positive and negative family history of cancer) than in controls. Plasma MDA levels in cervical cancer patients with a positive family history of cancer were greater (Table 6).

Correlation analysis between comet parameters and plasma MDA in cervical cancer cases and controls

In cervical cancer patients, there was a positive association between plasma MDA and two comet characteristics, such as comet tail length and DNA percentage in the comet head. Table 7 shows the correlation analysis between plasma MDA and comet parameters in both cases and controls.

**Table 7:** Correlation between plasma malondialdehyde with all comet parameters among cervical cancer cases and controls.

Plasma malondialdehyde levels	Comet parameters	Correlation coefficient (r)	Two-tailed significance (p-Value)
Cervical cancer patients	Total comet length (µm)	-0.132	0.367
	Comet head diameter (µm)	-0.314	0.028
	% of DNA in the comet head (%)	0.057	0.700
	Comet tail length (µm)	0.049	0.740
	% of DNA in the comet tail (%)	-0.099	0.498
Normal healthy controls	Total comet length (µm)	0.138	0.343
	Comet head diameter (µm)	0.168	0.248
	% of DNA the in comet head (%)	-0.046	0.752
	Comet tail length (µm)	-0.034	0.817
	% of DNA in the comet tail (%)	0.046	0.752

**Figure 2:**
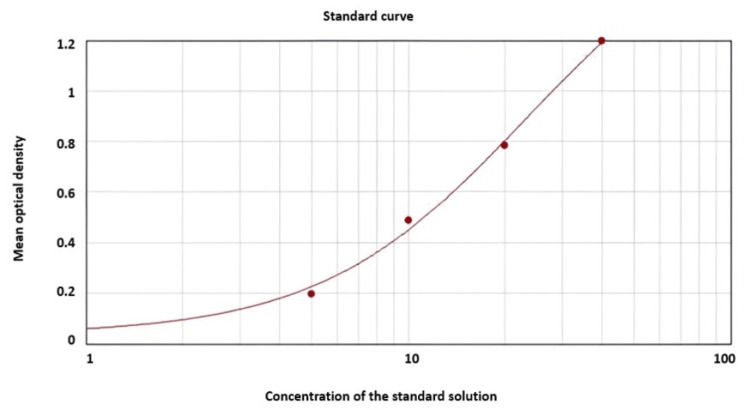
Standard curve obtained using the bioassay human MDA kit to analyze the plasma malondialdehyde levels. The standard curve is plotted against the x-axis and y-axis, where the x-axis represents the concentration of the standard solution and the y-axis represents the means of optical density of the standard solution.

Figure 2 shows the plasma malondialdehydes levels of cervical cancer cases followed the standard curve obtained using a bioassay human MDA kit.

**Figure 3:**
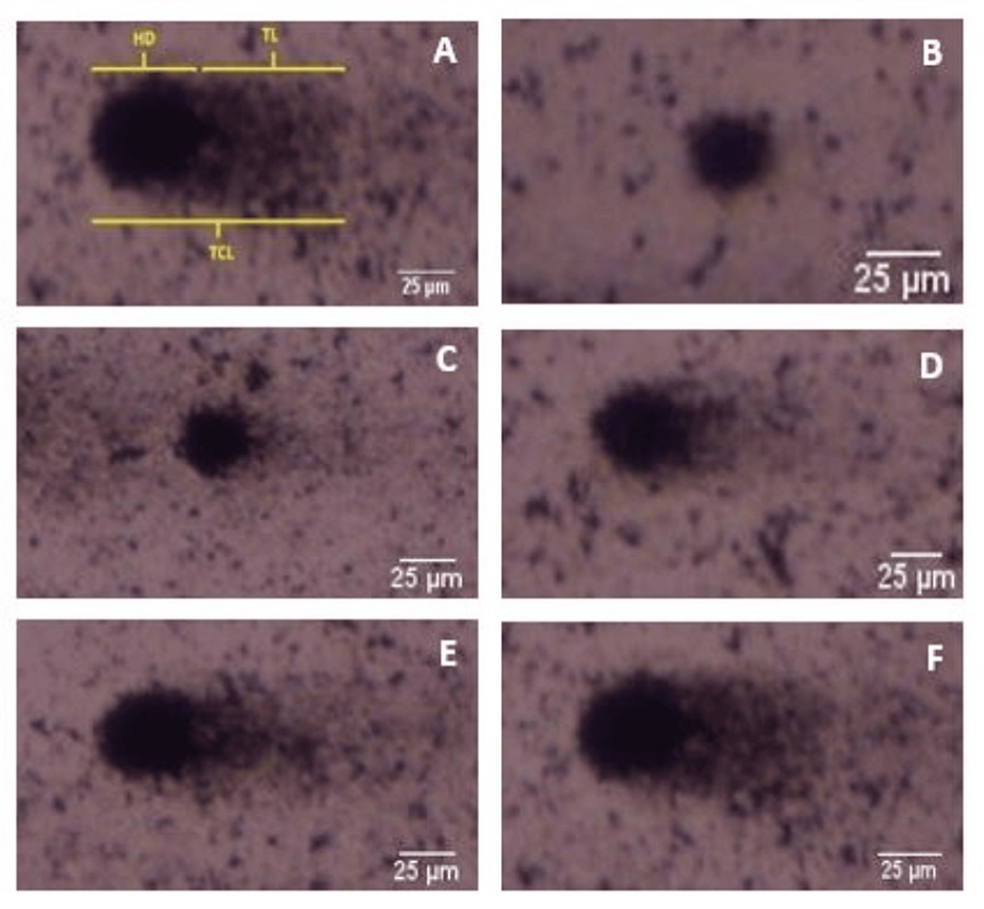
Photograph showing the parts of the comet and degree of DNA damage (0-4). A: Parts of the comet, B: 0-degree DNA damage, C: first-degree DNA damage, D: second-degree DNA damage, E: third-degree DNA damage, F: fourth-degree DNA damage. HD: head diameter, TL: tail length, TCL: total comet length.

Figure 3 depicts the sections of the comet recorded using an Olympus BX53 bright field microscope, as well as the degree of DNA damage from grade 0 to grade 4. 

**Figure 4:**
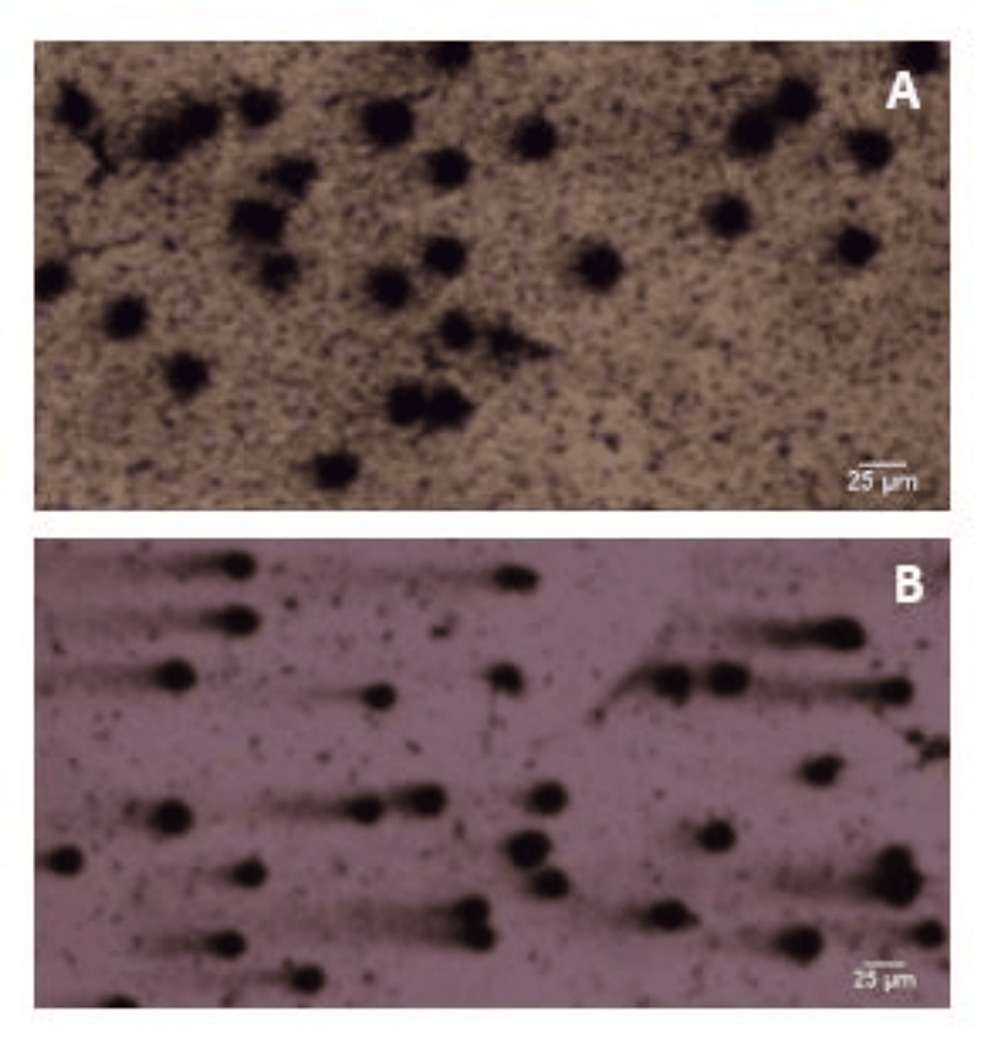
Photograph showing the comets of cervical cancer cases and controls. A: Comets of the controls (no migrated or damaged DNA seen), B: comets of cervical cancer patients (comets with migrated or damaged DNA seen along their tail length).

 Figure 4 depicts the comets seen in cervical cancer patients and controls.

## Discussion

Early detection and treatment of cervical cancer are critical for lowering death and morbidity. Various risk factors enhance free radical generation and disrupt the equilibrium between pro-oxidants and antioxidants. This imbalance causes an excess of free radicals to accumulate, causing damage to cellular elements such as lipids, carbohydrates, proteins, and nucleic acids (DNA), leading to genetic instability [36-40]. Cervical cancer is caused by such genetic instability over time [23,26,41,42]. The alkaline comet test can identify and quantify double-stranded DNA breakage. Parameters such as comet length, head diameter, percentage of DNA in the comet head, comet tail length, and percentage of DNA in the comet tail are utilized in alkaline comet tests to determine and quantify DNA damage. Excessive free radicals promote lipid peroxidation, which results in the formation of end products such as MDA [43,44].

Importance of comet parameters

Because of DNA damage, small DNA fragments tend to move away from the nucleoid bulk (comet head) during electrophoresis, increasing the length of the comet tail and the proportion of DNA in the comet tail. The dispersion of unmigrated nuclear material inside the comet head is indicated by the diameter of the comet head and the proportion of DNA in the comet head (undamaged DNA). The dispersion of migrated (damaged) DNA fragments in the comet tail is shown by the length of the comet tail, and the percentage of DNA in the comet tail reflects the number of migrated (damaged) DNA fragments inside the comet tail. As a result, the dispersion of migrated (damaged) DNA fragments in the comet tail and un-migrated (undamaged) DNA fragments in the comet head is indicated by the total comet length. Comet tail length (TL) and percentage of DNA in the comet tail (%T) were regarded as crucial indicators to evaluate the degree of DNA damage since these two measures reflect the exact amount of DNA damage [45,46].

Observations from the present study

We analyzed these comet characteristics and plasma MDA by categorizing research participants based on age, parity, number of pregnancies, risk factors, and cancer family history. Cervical cancer was shown to be more common in those over the age of 50, as well as in multiparous people, particularly those with two to four children. A positive family history of cervical cancer was found in 43% of cases and 6% of controls, emphasizing the significance of cancer family history and genetic susceptibility to cervical cancer. Early pregnancy (before the age of 19) was found in 57% of cervical cancer cases and 31% of controls, reinforcing the link between the early age of first pregnancy and cervical cancer. Early pregnancies, dysmenorrhea, a family history of cancer, drunkenness, and smoking were present in 75% of cervical cancer cases and 12% of controls, indicating a positive connection of risk factors in the etiology of cervical cancer. We analyzed these comet characteristics and plasma MDA by categorizing research participants based on age, parity, number of pregnancies, risk factors, and cancer family history. Cervical cancer was shown to be more common in those over the age of 50, as well as in multiparous people, particularly those with two to four children. A positive family history of cervical cancer was found in 43% of cases and 6% of controls, emphasizing the significance of cancer family history and genetic susceptibility to cervical cancer. Early pregnancy (before the age of 19) was found in 57% of cervical cancer cases and 31% of controls, reinforcing the link between the early age of first pregnancy and cervical cancer. Early pregnancies, dysmenorrhea, a family history of cancer, drunkenness, and smoking were present in 75% of cervical cancer cases and 12% of controls, indicating a positive connection of risk factors in the etiology of cervical cancer.

DNA damage in different age groups

We divided the research participants into four age groups: 30-39 years, 40-49 years, 50-59 years, and 60 years. Five comet parameters and plasma MDA levels were examined among cervical cancer patients and controls of various ages. Compared to controls, cervical cancer patients showed higher mean values for four comet characteristics, including comet length, tail length, percentage of DNA in the comet tail, head diameter, and plasma MDA levels. These differences were statistically significant. However, in the age range of 60 years, the comet head diameter was larger in cervical cancer cases than in controls, although the difference was statistically insignificant. In all age categories, the proportion of DNA in the comet head was higher in controls than in cervical cancer cases, and the difference between the two groups was statistically significant. The proportion of DNA in the comet head was also larger in controls than in cervical cancer cases in the age range of 60 years, but the difference between cervical cancer cases and controls was statistically insignificant. Thus, except for the head diameter and the proportion of DNA in the comet head, most comet characteristics were greater in cervical cancer cases than in controls across practically all age categories.

The comet parameters and plasma MDA levels in cervical cancer patients were examined across age groups. Comet parameters such as comet tail length, percentage of DNA in the tail, and plasma MDA levels increased in the 30-39 year age group. The head diameter increased in the 40-49 year age group, the percentage of DNA in the comet head increased in the 50-59 year age group, and the comet length increased in the 60-year age group. Three comet parameters and plasma MDA levels were raised in the 30-39 year age group among all comet parameters and plasma MDA levels. As a result, the risk of developing cervical cancer may be increased in the 30-39 age bracket compared to other age groups.

Sadeghi et al. found that women with no abnormalities, mild to moderate dysplasia, severe dysplasia, CIS, and invasive cervical cancer were on average 22.1, 22.8, 25.7, and 31.9 years old, respectively [47].

In our study of cervical cancer cases, we found that most of the DNA damage parameters were considerably higher in the 30-39 year age group compared to other age groups. Such an increase indicates a higher risk of cervical cancer in individuals aged 30-39 years. Conversely, Gandhi et al. found greater DNA damage in the 41-45 age range. Prabhakar et al. and Sobti et al. [48-50] found that the degree of DNA damage increases with age. However, our study did not observe such a trend.

Parity-wise DNA damage in cervical cancer

Participants in the research were divided into three parity groups: parity one, parity two to four, and parity five. Furthermore, we discovered that the parity distribution among research participants (both cervical cancer cases and controls) was unequal, with the majority of patients falling into the parity two to four range. As a result, we only evaluated the differences in cervical cancer cases and controls in parity groups of two to four.

Five comet parameters and plasma MDA levels were examined between cervical cancer patients and controls among parity groups. The median of four comet characteristics, including comet length, head diameter, tail length, percentage of DNA in the comet tail, and plasma MDA levels, was shown to be higher in cervical cancer cases than in controls with a parity of 2-4. There was a statistically significant difference between cervical cancer cases and controls. The proportion of DNA in the comet head was higher in controls than in cervical cancer cases with a parity of 2-4, and the difference between cases and controls was statistically significant. The majority of comet parameters and plasma MDA levels were greater in cervical cancer patients than in controls with two to four children.

Elizabeth et al. found a comparable distribution of research participants (cervical cancer cases and controls) into the parity group of two to four. Similar to our results, Elizabeth et al. observed that comet parameters were greater in cervical cancer patients compared to controls in the two to four parity group [51]. As a result, the chance of acquiring cervical cancer may be increased in the parity range of two to four when compared to other parity ranges. DNA damage was shown to be elevated in women who had many pregnancies, according to Gandhi et al. [48]. Prabhakar et al. and Sobti et al. grouped the parity groups as P1-3, P4-6, and P7, respectively, and discovered a significant variation in the comet test parameters between the parity groups. Excessive DNA damage was identified in persons with a larger number of pregnancies compared to those with a lower number of pregnancies, demonstrating that the DNA damage increased as the number of pregnancies rose [49,50].

DNA damage in early pregnancy

We divided the research participants into two groups based on their age at first pregnancy: (1) first pregnancy before the age of 19 and (2) first pregnancy after the age of 19. Five comet parameters and plasma MDA levels were compared between the two groups of cervical cancer patients and controls. The median of three comet characteristics, including comet length, comet tail length, and percentage of DNA in comet tail, was shown to be higher in cervical cancer cases compared to controls in category 1 (age 19 at first pregnancy), and the difference was statistically significant. However, when cervical cancer patients were compared to controls, the comet head diameter was larger, while the difference was statistically insignificant. The proportion of DNA in the comet head was higher in controls than in category 1 cervical cancer patients (age 19 at first pregnancy), and the difference was statistically significant.

When we analyzed the comet parameters and plasma MDA levels in two categories of cervical cancer patients, we discovered that the majority of the comet parameters that indicate DNA damage were found to be higher in category 1 instances compared to category 2 cases, and the difference was statistically significant. When compared to controls (n=15), a greater proportion of cervical cancer cases (n=28) fell into group one. Furthermore, data suggest that a person with an early pregnancy has a greater chance of acquiring cervical cancer. Similarly, Kurl et al., Sobti et al., and Edebiri et al. found that persons who married at a young age had a higher risk of developing cervical cancer [50,52,53]. Gandhi et al. discovered that in cervical cancer patients who were pregnant at a young age, DNA damage was elevated (14-16 years). He split the early pregnancies into two groups (14-16 and 17-19 years) and discovered a substantial increase in DNA damage in individuals with early pregnancy (14-16 years) [48].

Effects of risk factors

Participants in the research were divided into two groups based on their risk factors, with group 1 having less than or equal to one risk factor and group 2 having more than one risk factor. Furthermore, we discovered that the risk factor classification of cervical cancer cases and controls was unequally distributed. When cervical cancer cases were compared to controls with more than one risk factor, the median of three comet characteristics, such as comet length, tail length, and percentage of DNA in comet tail, rose. Furthermore, the difference between cases and controls was statistically significant. The comet head diameter and plasma MDA levels were greater in cervical cancer cases than in controls who had the risk variables more than once, although the difference was statistically insignificant. When compared to cervical cancer patients with more than one risk factor, the proportion of DNA in the comet head rose in controls. Furthermore, there was a statistically significant difference between cervical cancer cases and controls. As a result, except for the head diameter and percentage of DNA in the comet head, most comet characteristics were greater in cervical cancer cases than controls among persons who had more than one risk factor.

When we compared comet parameters and plasma MDA levels in cervical cancer cases with less than or equal to one risk factor and cervical cancer cases with more than one risk factor, we found that most of the parameters that denote DNA damage were increased in cervical cancer cases with more than one risk factor when compared to cervical cancer cases with one risk factor, and the difference was statistically significant. A greater proportion of cervical cancer cases (n=35) were found to have more than one risk factor than controls (n=6), indicating that the probability of acquiring cervical cancer is higher in those who have more than one risk factor. Gandhi et al. discovered that DNA damage was enhanced in cervical cancer patients with risk factors such as multiparity, early pregnancies, and poor socioeconomic level, i.e., those with more than one risk factor [48]

Effect of family history of cancer

When we compared the comet parameters and plasma MDA levels in cervical cancer cases and controls based on a family history of cervical cancer, we discovered that the median of three comet parameters, such as comet length, comet tail length, and percentage of DNA in comet tail, was found to be higher in cervical cancer cases compared to controls with a positive family history of cervical cancer, and the difference between the cervical cancer cases and controls was found to be higher in cervical cancer cases. However, plasma MDA levels were greater in cervical cancer cases than in controls with a positive family history of cervical cancer, and the difference between the cases and controls was statistically significant. The proportion of DNA in the comet head was higher in controls than in cervical cancer patients with a positive family history of cervical cancer, and the difference between the two groups was statistically significant. The comet head diameter was larger in controls than in cervical cancer patients, although the difference was statistically insignificant. Except for the comet head diameter and percentage of DNA in the comet head, the majority of comet parameters were greater in cervical cancer cases than in controls in persons with a positive family history of cervical cancer.

Zoodsma et al. discovered that persons with a positive family history of cervical cancer, particularly first-degree female relatives of cancer cases, had a greater chance of acquiring the disease than controls [54]. When we compared the comet parameters and plasma MDA levels in cervical cancer cases with and without a family history of cervical cancer, we discovered that most of the comet parameters that denote DNA damage were found to be higher in cervical cancer cases with a positive family history when compared to cervical cancer cases with a negative family history, and the difference was statistically significant. When compared to the controls, a larger proportion of cervical cancer cases (21 cases, 42%) were found to have a positive family history of cervical cancer (3 controls, 6%). A higher score implies that those with a positive family history of cervical cancer are at a greater risk of acquiring the disease. Similarly, Fischer et al. observed a similar result in the German population, Brinton et al. in the American population, and Furgyik et al. in the Swedish population [55-57].

The total comet length, comet head diameter, comet tail length, percentage of DNA in the comet tail, and plasma MDA were significantly higher in cervical cancer cases than in controls, except for the percentage of DNA in the comet head, which was statistically higher in controls than in cervical cancer cases. Similarly, Udumudi et al., Carlos Alvarez-Moya et al., and Gandhi et al. found that cervical cancer patients exhibited higher levels of DNA damage (comet tail length) than age-matched controls. Udumudi et al. and Carlos Alvarez-Moya et al. studied DNA damage in different stages of cervical cancer and discovered that DNA damage increased (comet tail length) as the stage of cervical cancer grew [34,48,58]. Smitha et al. investigated the impact of MDA, an oxidative stress marker, in cervical cancer patients. They examined plasma MDA in cervical cancer patients and compared it to age-matched controls and discovered a substantial rise in plasma MDA in cervical cancer patients compared to controls [59]. Smitha et al. discovered that a rise in plasma MDA was associated with a reduction in antioxidant levels (SOD, Vit C, and zinc) in cervical cancer patients [59].

Correlation analysis between plasma MDA and comet parameters in both cervical cancer cases and controls

In both cervical cancer cases and controls, correlation analysis was performed between plasma MDA levels and numerous comet parameters. Table 7 and Figure 1 show the correlation coefficient (r-value) and two-tailed significance (p-value) calculated from the data. Plasma MDA levels in cervical cancer patients were shown to be favorably connected with comet tail length and percentage of DNA in the comet head, and negatively (reversely) correlated with total comet length, percentage of DNA in the comet tail, and head diameter. Plasma MDA levels in controls were shown to be favorably connected with total comet length, percentage of DNA in the comet tail, and head diameter, and negatively linked with comet tail length and percentage of DNA in the comet head.

Based on the results presented above, we may conclude that factors that were favorably connected with plasma MDA in cervical cancer cases were negatively correlated with plasma MDA in controls (comet tail length and percentage of DNA in comet head). In cervical cancer patients, the characteristics that were adversely connected with plasma MDA were favorably correlated with plasma MDA in controls (total comet length, percentage of DNA in the comet tail, and head diameter). In instances of cervical cancer, the two essential comet characteristics that indicate increased DNA damage, comet tail length and percentage of DNA in the comet tail, should be raised in addition to plasma MDA levels. However, we discovered that increasing plasma MDA levels simply extended the length of the comet tail. Although the current research found a positive link between comet tail length and plasma MDA, the findings were statistically negligible, and the correlation between plasma MDA levels and comet characteristics was statistically insignificant in controls. Previous research, however, found a favorable relationship between comet tail length and plasma MDA levels [60,61]. Gao et al. investigated the impact of DNA damage in 45 female mice by inducing oxidative stress with environmental carcinogens (Benzopyrene). He discovered that increasing the dosage of a carcinogen increased plasma MDA levels and DNA damage (comet tail length) [61].

Similarly, Beevi et al. investigated 45 newly diagnosed instances of cervical squamous cell carcinoma. They discovered that oxidative stress indicators such as plasma MDA, nitric oxide, nitrite, and nitrate were elevated in cervical cancer patients. Cervical cancer patients had lower levels of antioxidant indicators such as erythrocyte superoxide dismutase, catalase, glutathione peroxidase, and glutathione transferase. Finally, Beevi et al. reported that lipid peroxidation indicators and nitric oxide products were elevated in cervical cancer patients, indicating an imbalance in the oxidant-antioxidant system [60]. Smitha et al. [59] discovered an increase in oxidative stress status (plasma MDA levels) as cervical cancer progressed. Despite having a sample size comparable to earlier research, we discovered that only the comet tail length was positively linked with plasma MDA levels, although statistically insignificantly. The proportion of DNA in the comet tail, on the other hand, demonstrated no positive connection with plasma MDA levels in cervical cancer patients or controls.

The current study's evidence for a positive association between comet tail length and plasma MDA levels is unclear. These equivocal findings might be attributed to the current study's lower sample size. Any uncertainty may be removed if the relationship between plasma MDA and comet properties is explored in a bigger sample size. It is critical to establish a positive or negative association between plasma MDA levels and the two most relevant comet test parameters (comet tail length and percentage of DNA in the tail). The relationship between plasma MDA and comet parameters emphasizes the importance of HPV infection.

DNA damage and accumulation of genetic instability

HPV-Induced Carcinogenesis

Chronic inflammation generated by viral oncogenes E5, E6, and E7 in HPV infections causes oxidative stress. This oxidative stress, in turn, will produce persistent inflammation and cellular damage by stimulating more inflammation and the production of different chemicals that cause cell damage and, eventually, carcinogenesis [26]. Tumor suppressor genes such as E6 and E7 oncoproteins inhibit the P53 and RB genes, respectively. The P53 and RB genes are suppressed by the nitric oxide generated during oxidative damage. The P53 and RB genes are both involved in cell cycle regulation. Impairment of these will disrupt the cell cycle, resulting in carcinogenesis [26]. The inflammation generated by HPV oncoproteins produces cytokines and growth factors, hastening the progression of cancer. HPV infections cause the activator protein-1 transcription factor to release E5, E6, and E7 oncoproteins (AP-1). This activator protein-1 promotes the production of cyclooxygenase-2 and prostaglandins in cancer cells, resulting in cell proliferation, angiogenesis, and reduced apoptosis [26].

Lipid peroxidation and generation of MDA

The oxidative stress generated by HPV infection disrupts redox equilibrium, eventually promoting chronic inflammation in cells. The lipids in the cell membrane are especially vulnerable to oxidative stress. The double bond between the two carbon atoms of PUFA is weakened by reactive oxygen species. The weaker link between carbon and hydrogen atoms permits the free radicals to liberate a hydrogen atom. Because of the release of a hydrogen atom from free radicals, lipid-free radicals are created, which are subsequently oxidized to make lipid peroxyl radicals, which in turn produce lipid hydroperoxide. Lipid hydroperoxide is an unstable molecule that breaks down to form MDA and four hydroxynonenals [27,28].

In HPV infection, the E5, E6, and E7 oncoproteins are responsible for two outcomes. One, they disrupt the cell cycle, causing DNA fragmentation and, eventually, carcinogenesis. Two, they promote oxidative stress and lipid peroxidation, resulting in higher levels of MDA [26]. As a consequence, any increase in DNA damage caused by carcinogens should result in a rise in plasma MDA levels. In the current investigation, plasma MDA levels in cervical cancer patients were found to be considerably higher than in controls. Similarly, when cervical cancer patients were compared to controls, there was a substantial increase in DNA damage. However, when just the cervical cancer cases are included, there is a modest positive link. This is a conundrum that requires additional examination with a larger sample size. Regardless of whether there is a positive or negative link between DNA damage and plasma MDA, we have seen a large rise in the comet characteristics of cervical cancer cases, which may be enough to infer that there is considerable DNA damage in cervical cancer cases compared to controls. Because of this, we may utilize the comet test (to assess DNA damage) as one of the instruments for screening cervical cancer patients. Similarly, plasma MDA levels in cervical cancer patients are much higher than in controls. This discovery also allows us to utilize the calculation of plasma MDA levels (lipid peroxidation) for cervical cancer screening.

PAP smear, VIAA, VILI, and HPV DNA tests are being utilized as screening procedures for the early detection of cervical cancer (precancerous stage) [34,42]. The current research sought to determine if there is considerable DNA damage in newly diagnosed cervical cancer patients before treatment initiation as compared to controls. When comparing cervical cancer patients to controls, we discovered that there was considerable DNA damage. As a result, the evaluation of DNA damage in peripheral blood cells using the comet assay and the calculation of plasma MDA using the TBARS technique might be utilized as a screening test to supplement the current cervical cancer screening methods. In essence, the estimation of DNA damage by comet assay and plasma MDA by TBARS assay may serve as a positive predictive tool for screening cervical cancer, particularly in individuals aged 30-39 years, with parity of two to four, and a history of early age at first pregnancy with a positive family history of cervical cancer.

Limitations

The generalizability of results may be restricted owing to the small sample size of 49 cervical cancer cases and 49 age-matched controls in the research. A larger and more diverse sample might increase the external validity of the results. The potential to analyze temporal changes or demonstrate causality is limited by the cross-sectional design. Confounding variables and selection bias, such as lifestyle characteristics and environmental exposures, might lead to confirmation of the reported relationships.

Strengths

The strengths of the study lie in its comprehensive approach to assessing DNA damage in patients diagnosed with cervical cancer and comparing these findings with age-matched controls. The utilization of the comet assay to measure DNA damage parameters, including comet length, head diameter, percentage of DNA in the comet head, tail length, and percentage of DNA in the comet tail, provides a detailed and multi-faceted evaluation of DNA damage. This method allows for a thorough understanding of the extent of DNA damage in cervical cancer patients compared to controls. Additionally, the inclusion of the oxidative stress marker, plasma MDA, provides valuable insight into the potential association between oxidative stress and DNA damage in the context of cervical cancer. The study's use of the TBARS ELISA method to measure plasma MDA levels ensures a standardized and reliable assessment of oxidative stress, enhancing the credibility of the findings. The positive correlation observed between plasma MDA and comet tail length further strengthens the study's conclusions, supporting the link between oxidative stress and DNA damage in cervical cancer patients. Furthermore, the identification of specific risk factors associated with increased DNA damage, such as age, parity, age at first pregnancy, and family history of cervical cancer, adds depth to the study's findings. This information can contribute to the development of targeted screening and risk assessment strategies for individuals at higher risk of developing cervical cancer. 

Future scope

The future scope of the study lies in its potential implications for clinical practice. The predictive value of the comet assay and plasma MDA estimation in identifying and assessing the risk of cervical cancer in specific demographic groups, such as individuals aged 30-39 with specific reproductive histories and familial predispositions, suggests the possibility of integrating these assessments into existing screening protocols. This could lead to more personalized and effective early detection and risk assessment strategies for cervical cancer.

## Conclusions

In our study, we observed a statistically significant difference in the comet test parameters in cervical cancer cases against controls, indicating a considerable increase in DNA damage in cervical cancer cases over controls. Similarly, there was a substantial rise in plasma MDA levels in cervical cancer patients compared to controls, indicating enhanced lipid peroxidation and oxidative stress. Individuals aged 30-39 years with a parity of two to four and a history of early age at first pregnancy with a positive family history of cervical cancer showed the most DNA damage compared to age-matched controls.
